# Books Reviewed and Books Received

**Published:** 1887-11

**Authors:** 


### BOOKS REVIEWED AND BOOKS RECEIVED.

OPERATIVE SURGERY ON THE CADAVER. JASPER JEWETT, GERMANY. Small 8vo, Pp. 150, cloth. NEW YORK: D. APPLETON & COMPANY.

A hand-book, such as this, was needed both by medical students and by graduates in medicine. It will be a useful book. The author has adopted the most recent nomenclature. The arrangement of the work is synoptical and systematic, and the land-marks given are accurate. The absence of illustrations of the operations and anatomical data regarding the parts involved detracts from the value of the work, which must nevertheless be considered a practical little book. The typographical work is excellent, and without error.

A PRACTICAL TREATISE ON MATERIA MEDICA AND THERAPEUTICS. By ROBERTS BARTHOLOW, M. A., M. D., LL. D. Sixth Edition. Revised and enlarged. Pp. xxiv. and 802. NEW YORK: D. APPLETON & Co., 1887. CHICAGO: W. T. KEENER.

The additions to this well-known treatise which have been made in this edition consist chiefly in a discussion of the large number of new remedies contributed by the chemical domain of pharmacology. Such recent drugs as Acetanilid, Pyndin, Urethan and others of their several classes, being classified according to their physiological action, and discussed in the author's characteristic and terse style. The new matter has added about one hundred pages to the treatise.

### BOOKS RECEIVED.

OUTLINES FOR THE MANAGEMENT OF DIET. By EDWARD TUNNIS BRUEN, M. D.

WHAT TO DO IN CASES OF POISONING. By WILLIAM MURREL, M. D., F. R. C. P.

EIGHTEENTH ANNUAL REPORT OF THE STATE BOARD OF HEALTH OF MASSACHUSETTS.

A MANUAL OF TREATMENT BY MASSAGE AND METHODICAL MUSCLE EXERCISE. By JOSEPH SCHREIBER, M.D. Translated by WALTER MENDELSON, M.D.

TRANSACTIONS OF THE THIRTY-SEVENTH ANNUAL MEETING OF THE ILLINOIS STATE MEDICAL SOCIETY, CHICAGO, MAY, 1887.

SYPHILIS. By JONATHAN HUTCHINSON, F. R. S., LL. D.

SURGERY, ITS THEORY AND PRACTICE. By WILLIAM JOHNSON WALSHAM, F. R. C. S.

THE STUDENT'S GUIDE TO DISEASES OF THE EYE. By EDWARD NETTLESHIP, F. R. C. S.

DIFFERENTIAL DIAGNOSIS: A MANUAL OF COMPARATIVE SEMEIOLOGY OF THE MORE IMPORTANT DISEASES. By F. DE HAVILLAND HALL, M. D.

THE PRINCIPLES OF ANTISEPTIC METHODS APPLIED TO OBSTETRIC PRACTICE. By DR. PAUL BAR. Translated by HENRY D. FRY, M. D.

A HANDBOOK OF GENERAL AND OPERATIVE GYNÆCOLOGY. By DR. A. HEGAR and DR. R. KALTENBACH. Edited by E. H. GRANDIN, M. D. Vol. VI. of Cyclopedia of Obstetrics and Gynæcology.

DISEASES OF THE FEMALE URETHRA AND BLADDER. By F. WINCKLE, M. D.; and DISEASES OF THE VAGINA. By A. BREISKY, M. D. Edited by EGBERT H. GRANDIN, M. D. Vol. X. of Cyclopedia of Obstetrics and Gynæcology.

DRUITT'S SURGEON'S VADE-MECUM. A MANUAL OF MODERN SURGERY. Edited by STANLEY BOYD, M. B., B. S., LOND., F. R. C. S. ENG.

DIFFERENTIAL DIAGNOSIS OF THE DISEASES OF THE SKIN. For Students and Practitioners. By CONDICT W. CUTTER, M. S., M. D.

VASO-RENAL CHANGE *versus* BRIGHT'S DISEASE. By J. MILNER FOTHERGILL, M. D., EDIN.

INDEX CATALOGUE OF THE LIBRARY OF THE SURGEON-GENERAL'S OFFICE, U. S. ARMY, AUTHORS AND SUBJECTS. Vol. VIII. LEGREN-MEDICINE. (Naval.)

